# Feasibility of Intersectoral Collaboration in Epidemic Preparedness and Response at Grassroots Levels in the Threat of COVID-19 Pandemic in Vietnam

**DOI:** 10.3389/fpubh.2020.589437

**Published:** 2020-11-17

**Authors:** Huong Thi Le, Hue Thi Mai, Hai Quang Pham, Cuong Tat Nguyen, Giang Thu Vu, Dung Tri Phung, Son Hong Nghiem, Bach Xuan Tran, Carl A. Latkin, Cyrus S. H. Ho, Roger C. M. Ho

**Affiliations:** ^1^Institute for Preventive Medicine and Public Health, Hanoi Medical University, Hanoi, Vietnam; ^2^Institute for Global Health Innovations, Duy Tan University, Da Nang, Vietnam; ^3^Faculty of Medicine, Duy Tan University, Da Nang, Vietnam; ^4^Center of Excellence in Evidence-Based Medicine, Nguyen Tat Thanh University, Ho Chi Minh City, Vietnam; ^5^School of Medicine, Griffith University, Brisbane, QLD, Australia; ^6^Centre for Applied Health Economics (CAHE), Griffith University, Brisbane, QLD, Australia; ^7^Bloomberg School of Public Health, Johns Hopkins University, Baltimore, MD, United States; ^8^Department of Psychological Medicine, National University Hospital, Singapore, Singapore; ^9^Department of Psychological Medicine, Yong Loo Lin School of Medicine, National University of Singapore, Singapore, Singapore; ^10^Institute for Health Innovation and Technology (iHealthtech), National University of Singapore, Singapore, Singapore

**Keywords:** COVID-19, intersectoral collaboration, epidemic preparedness, grassroots level, Vietnam

## Abstract

To effectively control the COVID-19 (coronavirus disease 2019) outbreak in later stages in Vietnam requires addressing the existing gaps in the national health emergency framework, consolidate, and inform its structure, we conducted this study to evaluate the importance and collaborative mechanism between health and community service workers with intersectional organizations at grassroots levels in Vietnam. A cross-sectional, web-based survey was conducted from 12/2019 to 02/2020 on 581 participants (37 health workers, 473 medical students, and 71 community service workers). The snowball sampling technique was used to recruit participants. We used exploratory factor analysis to test the construct validity of the questionnaire measuring the perceived efficiency of involving community service workers in health care–related activities and Tobit models to examine its associated factors. The results showed the importance of local organizations in epidemic preparedness and response at grassroots levels, with scores ranging from 6.4 to 7.1, in which the Vietnam Youth Federation played the most important role (mean = 7.1, SD = 2.2). Of note, community service workers were viewed as performing well in health communication and education at agencies, schools, and other localities. Medical students perceived higher efficiency of involving community service workers in health care–related activities at grassroots levels as compared to health workers. We encourage the government to promote intersectoral collaboration in epidemic preparedness and response, giving attention to scale up throughout training as well as interdistrict and interprovincial governance mechanisms.

## Introduction

Originating from Hubei province in China, coronavirus disease 2019 (COVID-19) dramatically spread to many parts of the world (1, 2). As of May 11, 2020, there were 4.01 million confirmed cases reported globally, including 88,891 new cases and 278,892 fatal cases, covering 216 countries and territories (3). Understanding the severity and coverage of a pandemic, the World Health Organization has circulated strategic preparedness and response plan for COVID-19 (4). The overall goal of this plan is to stop further COVID-19 transmission and minimize the impacts in all nations. Accordingly, there are three core strategies that can flexibly tailor to a particular context of each country, including (1) establishing international coordination and operational support (2), promoting preparedness and response at the national level, and (3) accelerating research and innovation (4).

While much of the world has recognized the integral role of preparedness and response in the epidemic mitigation, each country has currently developed its own context-specific strategy, which is driven by various factors such as socioeconomic features, health system, and existing resources. Some countries with well-integrated health system and strong human resources, such as the Netherlands and Sweden, have tried to build rapid herd immunity, which occurs when most of the community has become immune to COVID-19 so that it could indirectly protect the community from the infection (5, 6). However, this approach immediately consumes enormous resources and has raised the world concern about its effectiveness because of the drastic increase in the number of COVID-19 cases and deaths in these countries (7). Thus, it perhaps would not be the best-fit model for low-resource settings such as Vietnam, a developing country with a population of more than 100 million people and sharing more than 1,200 km of the border with China.

Instead, Vietnam has currently implemented a strategy of early detection of COVID-19 infection, effective isolation and timely treatment for confirmed cases, and minimizing deaths; at the same time, promoting disease prevention (8, 9). The plan has shown excellent results in the early stages of fighting the COVID-19 epidemic. So far, Vietnam has a much lower number of confirmed cases compared to other neighboring countries, with 239 confirmed cases and no deaths reported by April 2020 (2, 9). However, as the unprecedented and unpredictable spread of COVID-19, it seems to be too early to conclude the successful containment of the outbreak in Vietnam. To maximize the capacity to prevent an uncontrollable outbreak, the existing gaps in the national health emergency framework, as indicated by the Self-Assessment Annual Reporting tool 2018 (10), should be immediately addressed. This requires the quick mobilization of existing resources other than health sectors such as community units and organizations, especially at the grassroots levels where human resources for health are under severe constraints, and the mobilization and management process will be much more effective under the direction of local organizations instead of at the central level (11). In order to consolidate and inform the national health emergency framework, which is responsible for the containment of the outbreak in later stages in Vietnam, we conducted this study to evaluate the importance and collaborative mechanism between health and community service workers with intersectional organizations at grassroots levels in epidemic preparedness and response.

## Materials and Methods

### Study Design and Setting

We conducted a cross-sectional study from 12/2019 to 02/2020 in all provinces of Vietnam via online platforms named SurveyMonkey. SurveyMonkey is one of the most popular online survey sites, due to its intuitive and easy to share. The system allows researchers to create questionnaires with a variety of types of questions and still ensure security by password management system and personal email.

### Study Subject

We recruited all health workers, medical students, and community workers through the country if they met the following criteria: (1) being at least 18 years old (2), currently living in Vietnam, and (3) agreeing to participate in the study. Those who were cognitively impaired were excluded from the study. All participants were asked to provide informed consent to confirm their voluntary participation by clicking “agree to participate in the study” after reading the study purposes, their benefits, and responsibilities.

### Sampling Procedure and Sample Size

We recruited the exponential non-discriminative snowball sampling technique to recruit respondents using online platforms. This method is considered as a cost-effective, non-probability method that allowed us to locate hidden populations by relying on referrals from one initial respondent to other potential respondents (12). Also, a web-based survey is suitable and effective in the current context, because due to the tremendous impact of the COVID-19 outbreak, people tend to work frequently online; therefore, it allowed us to reach a large number of participants. We sent the questionnaire link to the target participants via email and Facebook. These are the most commonly used online platforms in Vietnam. We recruited 37 health workers, 473 medical students, and 71 community workers. The total sample size was 581 participants.

### Study Instruments and Measures

To collect data, we developed a self-reported questionnaire with three main following sections:

#### Sociodemographic Characteristics

Sociodemographic characteristics included age, gender, living area (urban or rural), marital status (single, married, etc.), education level, having participated in youth association activities, workplace level (central level, provincial level, under provincial level, or working for university).

#### The Perceived Importance of Community Workers in Health Care, Epidemic Preparedness, and Response at Grassroots Levels

We asked the participants to rate the importance of organizations in health care, epidemic preparedness, and response at grassroots levels. These organizations included the following:

- Ho Chi Minh Communist Youth Union: the largest social–political organization of Vietnamese youth
- Vietnam Youth Federation: broad social organization of Vietnamese youths and youth organizations
- The Vietnamese Fatherland Front: a political coalition organization, a voluntary union of political organizations, sociopolitical organizations, social organizations and individuals representing all classes, social strata, ethnic groups, religions, and overseas Vietnamese
- Viet Nam Farmer's Union: a social–political organization of Vietnamese peasantry
- Viet Nam Women's Union: a sociopolitical organization that represents and defends the legal and legitimate rights and interests of Women in Vietnam
- Local occupational associations: organizations that protect the legal and legitimate rights and interests of their members within the legal framework for a certain profession in the locality
- Religious and belief units: organizations that have the task of regulating, protecting and being the voice of people following different religions, beliefs before the law because Vietnam allows people to freely choose their religion
- Non-governmental organizations: non-profit, citizen-based groups that function independently of government and has an important role to play in supporting resources
- Social businesses: organizations that apply commercial strategies to maximize improvements in financial, social, and environmental well-being.

There were 10 levels of response in each question, with a higher score indicating greater importance.

### The Perceived Efficiency of Involving Community Service Workers in Health Care–Related Activities

Participants rated the levels of efficiency of involving community service workers in health care–related activities, including the following: (1) identify the risks of environmental pollution, changes in natural conditions, farming, and unusual weather phenomena; (2) report weather phenomena, pollution risks to the community and authorities; (3) detect and promptly notify disease risks and new cases to the locality; (4) participate in local epidemic prevention and response; (5) participate in health care and improving the health of people who affected by the epidemics, natural disasters such as floods and droughts; (6) guide and support people with abnormal health signs to go to health facilities; (7) participate in health communication and education at agencies, schools, and localities; (8) participate in stabilizing life, livelihood, and local security before, during, and after natural disasters and epidemics. There were 10 levels of response in each question, with a higher score indicating greater efficiency.

### Data Management and Analysis

Data were collected by SurveyMonkey and automatically saved in the system. Only approved members could access and export the data. We analyzed data with Stata (version 15, Stata Corp LP, College Station, TX, USA). Quantitative variables were summarized in mean and standard deviation (SD). The differences between these variables were tested using Kruskal–Wallis test, with *p* < 0.05 considered statistically significant. We utilized frequency and percentage to describe qualitative variables. Fisher exact test and χ^2^ test were used to test the differences between these variables. We used exploratory factor analysis to explore the construct validity of the questionnaire measuring the perceived efficiency of involving community service workers in health care–related activities. Tobit model was applied to determine factors associated with perceived efficiency of involving community service workers in health care–related activities at grassroots levels. The independent variables, including socioeconomic status, occupation characteristics, and perceived importance of organizations, were entered in the full regression models. The study applied a stepwise forward selection to construct the reduced model that selected variables based on the log-likelihood ratio test at *p* < 0.2.

### Ethical Consideration

The study protocol was reviewed and approved by the Scientific Council of Vietnam Central Youth Union (No 177 QÐ/TWÐTN-VNCTN). Participation was completely voluntary, and there were no incentives provided. Collected data were saved in a secured system and only served for the study purposes.

## Results

Table 1 summarizes sociodemographic characteristics of respondents. In a total of 581 participants, 37 respondents were health workers, 473 respondents were medical students, and 71 respondents were community service workers. The number of females in the sample was twice that of males (31.2 and 68.9%, respectively). The majority were younger than 25 years (81.4%), lived in the urban (85.7%), were single (85.8%), and participated in community activities. The average age of participants was 22.6 (SD = 5.3) years, and there was a statistically significant difference in age among health workers, medical students, and community service workers (*p* < 0.01).

**Table 1:** Sociodemographic characteristics of respondents.

	**Health workers**		**Medical students**		**Community service workers**		**Total**		***p***
	***n***	**%**	***n***	**%**	***n***	**%**	***n***	**%**	
Total	37	6.4	473	81.4	71	12.2	581	100.0	
**Gender**									
Male	16	43.2	123	26.0	42	59.2	181	31.2	**<0.01**
Female	21	56.8	350	74.0	29	40.9	400	68.9	
**Living area**									
Urban	31	83.8	401	86.1	60	84.5	492	85.7	0.89
Rural	6	16.2	65	14.0	11	15.5	82	14.3	
**Marital status**									
Single	11	29.7	462	98.1	24	33.8	497	85.8	**<0.01**
Living with	26	70.3	1	0.2	44	62.0	71	12.3	
spouse									
Others	0	0.0	8	1.7	3	4.2	11	1.9	
**Workplace**									
Central level	5	13.5	52	11.2	16	23.9	73	12.8	**<0.01**
Provincial level	21	56.8	54	11.6	32	47.8	107	18.8	
< Provincial level	9	24.3	4	0.9	19	28.4	32	5.6	
College/university	2	5.4	355	76.3	0	0.0	357	62.7	
**Participated in community activities**									
Yes	31	83.8	197	41.7	71	100.0	299	51.6	**<0.01**
No	6	16.2	275	58.3	0	0.0	281	48.5	
**Age group**									
<25 years	3	8.6	448	98.9	3	4.3	454	81.4	**<0.01**
≥ 25 years	32	91.4	5	1.1	67	95.7	104	18.6	
	**Mean**	**SD**	**Mean**	**SD**	**Mean**	**SD**	**Mean**	**SD**	***p***
**Age**	32.0	6.9	20.5	1.5	32.0	4.8	22.6	5.3	**<0.01**

Table 2 illustrates the perceived importance of organizations in health care, epidemic preparedness, and response at grassroots levels. In general, respondents highly evaluated the importance of organizations in epidemic and response at grassroots levels (mean scores ranged from 6.4 to 7.1). Respondents rated Vietnam Youth Federation (mean = 7.1, SD = 2.2) and Ho Chi Minh Communist Youth Union (mean = 7.0, SD =2.3) as the most important in health care, epidemic preparedness, and response at grassroots levels. Meanwhile, religious and belief units (mean = 6.4, SD = 2.5) were the least important.

**Table 2:** The perceived importance of organizations in health care, epidemic preparedness, and response at grassroots levels (10-point scale).

	**Health workers**		**Medical students**		**Community service workers**		**Total**		***p***
	**Mean**	**SD**	**Mean**	**SD**	**Mean**	**SD**	**Mean**	**SD**	
Vietnam Youth Federation	6.1	2.3	7.2	2.1	6.9	2.9	7.1	2.2	**0.03**
Ho Chi Minh Communist Youth Union	6.4	2.6	7.1	2.1	6.8	2.9	7.0	2.3	0.33
The Vietnamese Fatherland Front	5.8	2.7	7.1	2.2	6.8	2.7	7.0	2.3	**0.02**
Viet Nam Farmer's Union:	6.0	2.7	7.1	2.2	6.7	2.7	7.0	2.3	**0.03**
Non-governmental organizations	6.6	2.4	7.0	2.4	6.6	2.4	6.9	2.4	0.32
Social businesses	6.1	2.4	7.0	2.2	6.6	2.5	6.9	2.2	0.05
Viet Nam Women's Union	5.8	2.5	7.1	2.1	6.6	2.8	6.9	2.3	**0.01**
Local occupational associations	5.7	2.5	7.0	2.2	5.9	2.9	6.8	2.4	**<0.01**
Religious and belief units	5.6	2.5	6.6	2.3	5.6	3.1	6.4	2.5	**<0.01**

*Bold values indicate significant p-value < 0.05*.

The construct validity of the questionnaire measuring the perceived efficiency of involving community service workers in health care–related activities at grassroots levels is described in Table 3. The series of questions were reclassified into two domains called “epidemic investigation, counseling, and control” and “reporting and monitoring environmental changes and health problems.” The Cronbach α was very high in both domains (0.95 and 0.91, respectively).

**Table 3:** Factor loadings of the questionnaire measuring the perceived efficiency of involving community service workers in health care–related activities at grassroots levels.

**Items**	**Totally effective**		**Epidemic investigation, counseling, and control**	**Reporting and monitoring environmental changes and health problems**
	***n***	**%**		
(1) Participate in health communication and education at agencies, xsschools, and localities	104	18.0	0.87	
(2) Guide and support people with abnormal health signs to go to health facilities	84	14.5	0.80	
(3) Report weather phenomena, pollution risks to the community and authorities	82	14.2		0.77
(4) Participate in local epidemic prevention and response	80	13.8	0.69	
(5) Participate in health care and improving the health of people who affected by the epidemics, natural disasters, floods, and droughts	78	13.5	0.84	
(6) Participate in stabilizing life, livelihood, and local security before, during, and after natural disasters and epidemics	75	13.0	0.65	
(7) Identify the risks of environmental pollution, changes in natural conditions, farming, and unusual weather phenomena	75	12.9		0.88
(8) Detect and promptly notify disease risks and new cases to the locality	68	11.8		0.76
**Cronbach's** **α**			0.95	0.91
**Mean**			7.3	6.9
**SD**			1.8	1.9

Table 3 showed the percentage of participants who perceived that the involvement of community service workers in health care–related activities at grassroots levels was highest in “Participate in health communication and education at agencies, schools, and localities” (18%), followed by “guide and support people with abnormal health signs to go to health facilities” (14.2%). In contrast, the percentage of participants who perceived that the involvement of community service workers in health care–related activities at grassroots levels was lowest in “detect and promptly notify disease risks and new cases to the locality” (11.8%), followed by “identify the risks of environmental pollution, changes in natural conditions, farming, and unusual weather phenomena” (12.9%).

As shown in Table 4, the mean score of the domain “epidemic investigation, counseling, and control” was 7.3 (SD = 1.8), and in the domain “reporting and monitoring environmental changes and health problems,” the mean was 6.9 (SD = 1.9). In the domain, “epidemic investigation, counseling, and control,” respondents rated “participate in health communication and education at agencies, schools, and localities” to be the most effective (mean = 7.5, SD = 1.9). In the domain, “reporting and monitoring changes in environment and health,” participants rated the highest score for “report weather phenomena, pollution risks to the community and authorities” (mean = 7.1, SD = 2.0).

**Table 4:** The perceived efficiency of involving community service workers in health care–related activities at grassroots levels.

**Evaluation of respondents on**	**Medical professional**		**Medical students**		**Community workers**		**Total**		***p* value**
	**Mean**	**SD**	**Mean**	**SD**	**Mean**	**SD**	**Mean**	**SD**	
**Epidemic investigation, counseling, and control**	7.0	1.5	7.3	1.7	7.0	2.0	7.3	1.8	0.18
Participate in local epidemic prevention and response	6.9	2.1	7.3	1.9	6.8	2.6	7.2	2.0	0.43
Participate in health care and improving the health of people who affected by the epidemics, natural disasters, floods, and droughts	6.7	1.8	7.3	1.9	7.1	2.3	7.3	1.9	0.14
Guide and support people with abnormal health signs to go to health facilities	7.0	1.7	7.4	1.9	6.8	2.4	7.3	2.0	0.11
Participate in health communication and education at agencies, schools, and localities	7.3	1.7	7.5	1.9	7.5	2.0	7.5	1.9	0.51
Participate in stabilizing life, livelihood, and local security before, during, and after natural disasters and epidemics	7.1	1.8	7.1	1.9	6.8	2.3	7.1	1.9	0.69
**Reporting and monitoring environmental changes and health problems**	6.2	1.9	7.0	1.8	6.6	2.3	6.9	1.9	**0.02**
Identify the risks of environmental pollution, changes in natural conditions, farming, and unusual weather phenomena	6.3	2.4	6.9	2.1	6.6	2.6	6.9	2.2	0.20
Report weather phenomena, pollution risks to the community and authorities	6.8	2.0	7.1	2.0	6.7	2.3	7.1	2.0	0.31
Detect and promptly notify disease risks and new cases to the locality	5.7	2.3	7.1	1.9	6.4	2.7	6.9	2.1	**<0.01**

*Bold values indicate significant p-value <0.05*.

Table 5 summarizes the results of the regression analysis. Those who lived with spouse tended to perceive the higher efficiency of involving community service workers in epidemic investigation, counseling and control [coefficient = 0.62, 95% confidence interval (CI) = 0.16–1.09], and reporting monitoring changes in environment and health (coefficient = 0.75, 95% CI = 0.16–1.34). Of note, medical students were more likely to perceive the efficiency of involving community service workers in reporting and monitoring changes in environment and health compared to health workers (coefficient = 0.75, 95% CI = 0.03–1.06). Of note, those who felt the higher importance of youth associations and social businesses perceived higher efficiency of involving community service workers in epidemic investigation, counseling and control, and reporting and monitoring changes in environment and health.

**Table 5:** Factors associated with perceived efficiency of involving community service workers in health care–related activities at grassroots levels.

	**Epidemic investigation, counseling, and control**		**Reporting and monitoring environmental changes and health problems**	
	**Coefficient**	**95% CI**	**Coefficient**	**95% CI**
**Marital status** (living with spouse vs. single)	0.62^***^	(0.16–1.09)	0.75^**^	(0.16–1.34)
**Objects** (vs. health workers)				
Medical students			0.55^**^	(0.03–1.06)
Community service workers	−0.41^*^	(−0.87 to 0.04)		
**Participated in community activities** (yes vs. no)	−0.21	(−0.48 to 0.05)	−0.25^*^	(−0.53 to 0.02)
**Perceived importance of organizations by respondents**				
(10-point scale)				
Vietnam Youth Federation	0.39^***^	(0.32–0.46)	0.30^***^	(0.21–0.40)
Local occupation associations			0.12^**^	(0.03–0.21)
Non-governmental organizations	0.10^***^	(0.03–0.18)	0.14^***^	(0.06–0.22)
Social businesses	0.18^***^	(0.09–0.26)	0.16^***^	(0.07–0.25)

*** *p < 0.01*,

** *p < 0.05*,

* *p < 0.1*.

## Discussion

### Key Findings

This study evaluated the importance and collaborative mechanism between health and community service workers with intersectional organizations at grassroots levels in epidemic preparedness and response. We found that local organizations were existing resources that could be immediately mobilized to address the epidemic; in which youth played a critical role. In the intersectoral mechanism, community service workers were best performed in health communication and education at agencies, schools, and localities. Of note, medical students perceived higher efficiency of involving community service workers in health care–related activities at grassroots levels as compared to health workers.

Facing the COVID-19 pandemic, even the most developed countries may not devote sufficient resources at the initial stages (13), and this situation may be even worse in low and middle developing countries (14). Thus, taking the advantages of on-site resources would be full of potential to scale up country readiness and response operations. In Vietnam, it is a long-held practice that health workers, including doctors, nurses, technicians, and traditional medicine practitioners, pharmacists, are responsible for all health issues of national and international concerns (15). However, overreliance on this specialized taskforce may fail to meet the drastic demand of the population in urgent cases, such as the COVID-19 outbreak. It is advisable, in such situations, to make prudent use of local organizations to ensure a sufficient workforce to respond quickly. In Vietnam, these include the Ho Chi Minh Communist Youth Union, Vietnam Youth Federation, The Vietnamese Fatherland Front, Farmers Association, Women Union, local occupation associations, religious and belief units, non-governmental organizations, and social businesses. These organizations can be actively involved in non–treatment-related activities such as risk communication, health education and surveillance, and aggressive contact tracing. Moreover, as each unit and organization are assigned to a particular task, they may help to inform the response framework and rapid action team at grassroots levels in particular and the national level as a whole.

In the intersectoral mechanism, we found that in Vietnam, the youth should be the priority for mobilization at grassroots levels. In Vietnam, young people aged 15–29 years account for 25% of the total population (16). More importantly, they are better educated (16) and more socially responsible compared to previous generations (17). Thus, in the context of accelerated national and global issues such as infectious disease outbreaks, climate change, and pollution, they could act as the vanguard in community- and social-based activities. At the moment, Vietnam has been mobilized thousands of medical students to support the COVID-19 response with various activities such as epidemiological investigation, quarantine guide, and blood testing (18). It has been critical to systematically scale up youth networks from local to central levels in antiepidemic activities. One of the most feasible tasks, which was indicated in our study, is health communication and education at agencies, schools, and localities. To achieve this, however, requires thorough training and proper interdistrict and interprovincial governance mechanisms.

As reported in our study, medical students perceived higher efficiency of involving community service workers in health-related activities at grassroots levels compared to health workers. Given the core role of medical students in community networks for the epidemic preparedness and response, their perspectives about the efficiency of engaging community service workers in health-related activities are insightful. Thus, it enriches the understanding of the efficiency and feasibility of the collaborative mechanism between health and community service workers with intersectional organizations at grassroots levels in Vietnam.

### Limitations

While the study results have provided a valuable contribution to the current understanding of intersectoral collaboration in epidemic preparedness and response at grassroots levels in the threat of COVID-19 pandemic in Vietnam, there are several limitations. An online-based survey may fuel the risks of survey fraud because it did not allow us to confirm participants' identities. To minimize such risks, we did not offer participants with incentives to eliminate the possibility of study participation for incentive purposes. Besides, we used the snowball sampling method, which may introduce sampling bias. This non-probability sampling could affect to the representativeness of the survey sample. Ideally, a random-sampling technique would minimize this risk; however, it would be costly and, in the current context, infeasible. Third, the study was conducted only in Vietnam, so that the implications would be questioned about their effectiveness and implications when applying in other settings, especially at developed countries. However, based on the fact that Vietnam has achieved outstanding results in COVID-19 prevention and the strength of the time of the study, we believe that these finding can serve as a reference for preparedness and response strategy in other countries.

## Conclusion

In conclusion, this study indicated the great potential and feasibility of intersectoral collaboration in epidemic preparedness and response at grassroots levels in the threat of COVID-19 pandemic in Vietnam. This approach will not only ensure sufficient resources for urgent cases but also help to inform and shape a determined response framework and rapid action team at the grassroots levels in particular and national levels as a whole. In this intersectoral mechanism, the youth can be the vanguard in community- and social-based activities, especially in health communication and education at localities. To achieve it requires throughout training, interdistrict, and interprovincial governance mechanisms.

## Data Availability Statement

The raw data supporting the conclusions of this article will be made available by the authors, without undue reservation.

## Ethics Statement

The studies involving human participants were reviewed and approved by The study protocol was reviewed and approved by the Scientific Council of Vietnam Central Youth Union (No 177 QÐ/TWÐTN-VNCTN). The patients/participants provided their written informed consent to participate in this study.

## Author Contributions

HL, GV, and BT designed and supervised the study. HM, HP, and CN collected, analyzed, and interpreted the data. HL, HM, and DP drafted the manuscript. SN, CL, CH, and RH reviewed the manuscript and commented on the writing. All authors agreed with the final manuscript.

## Conflict of Interest

The authors declare that the research was conducted in the absence of any commercial or financial relationships that could be construed as a potential conflict of interest.
